# Tonate Virus and Fetal Abnormalities, French Guiana, 2019

**DOI:** 10.3201/eid2802.210884

**Published:** 2022-02

**Authors:** Veronique Lambert, Antoine Enfissi, Mathilde Lefebvre, Leo Pomar, Sobhi Kedous, Fabien Guimiot, Gabriel Carles, Anne Lavergne, Dominique Rousset, Najeh Hcini

**Affiliations:** West French Guiana Hospital Center, Saint-Laurent-du-Maroni, French Guiana (V. Lambert, L. Pomar, S. Kedous, G. Carles, N. Hcini);; Institut Pasteur Arbovirus National Reference Center, Cayenne, French Guiana (A. Enfissi, A. Lavergne, D. Rousset);; Centre Hospitalo-Universitaire Robert Debre, Paris, France (M. Lefebvre, F. Guimiot);; University of French Guiana, Cayenne, French Guiana (N. Hcini)

**Keywords:** tonate virus, fetal abnormalities, pregnancy, vertical disease transmission, arboviruses, French Guiana, brain diseases, Venezuelan equine encephalitis, prenatal diagnosis, viruses, meningitis/encephalitis

## Abstract

We report a case of vertical transmission of Tonate virus in a pregnant woman from French Guiana. The fetus showed severe necrotic and hemorrhagic lesions of the brain and spinal cord. Clinicians should be made aware of possible adverse fetal outcomes in pregnant women infected with Tonate virus.

Venezuelan equine encephalomyelitis (VEE) complex viruses consist of antigenically related arboviruses widely distributed throughout the Americas (*1*). Only subtype I varieties AB and C cause severe equine epizootics and human outbreaks marked by the occurrence of encephalitis and fetal damage (*2*). The other subtypes are endemic in small areas of South America (*3*). In 1973, subtype III-B, the Tonate virus (TONV), was isolated in birds from French Guiana (*4*). It has since been found in neighboring countries and in South Dakota and Colorado in the United States (*5*,*6*). The wild cycle of TONV is still poorly understood. Transmission by Culicidae insects has been observed during the rainy season (*4*). Birds and bats are the only identified vertebrate hosts (*7*). In humans in French Guiana, TONV seroprevalence suggests endemic transmission, particularly along the coast of the Bas Maroni region (*8*). However, clinical descriptions remain scarce, and no adverse pregnancy outcomes or vertical transmission have been reported (*9*,*10*). We report a case of vertical transmission of TONV from a pregnant woman to her fetus and describe ultrasonographic and fetopathological findings. 

## The Study

During the 2019 rainy season, a 33-year-old woman living in the Bas Maroni region of French Guiana was referred to the prenatal diagnosis unit at West French Guiana Hospital Center (Saint-Laurent-du-Maroni, French Guiana) for fetal anomalies. This healthy G8P7 woman had no history of genetic disorders or birth defects from previous pregnancies. She was asymptomatic during the first trimester of pregnancy and tested negative for syphilis, toxoplasmosis, rubella, cytomegalovirus, chikungunya, and Zika. An ultrasound screening performed at 20 weeks of gestation showed a hydropic fetus with microcephaly. The atrophic cerebral mantle exhibited calcifications and moderate ventriculomegaly. The corpus callosum, the cerebellum, and the brain stem were dysplastic. The fetus manifested limb malformations and an absence of swallowing at the time of the serially performed sonograms (Appendix Figure; Video). Therefore, we performed amniocentesis for etiological investigation. Because of the poor prognosis, the mother elected to terminate the pregnancy. After approval by the multidisciplinary center for prenatal diagnosis, the pregnancy was terminated without complication. The patient gave written informed consent for the publication of her case.

**Video:** Ultrasonographic prenatal imaging of fetus with developmental abnormalities.

Karyotype and array comparative genomic hybridization were normal. Results of screening for metabolic diseases were negative. All PCR and reverse transcription PCR (RT-PCR) for toxoplasmosis, rubella, cytomegalovirus, herpes simplex virus, and common arboviruses from the Amazon were negative. However, we reproducibly detected the presence of a VEE complex virus in the amniotic fluid with a real-time RT-PCR test yielding cycle threshold values of 30. Furthermore, although maternal serum samples collected 2 months before pregnancy were negative for TONV IgM, the test was positive at the time of pregnancy termination.

**Figure 1:**
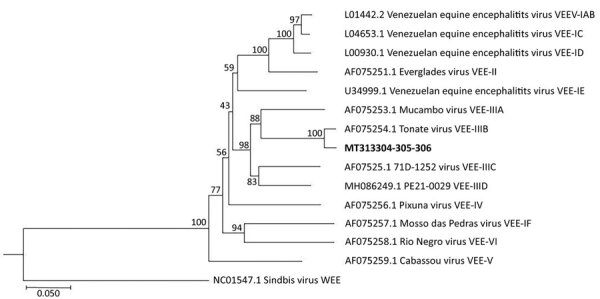
Phylogenetic tree of VEE complex viruses showing close relationship between a virus from the amniotic liquid of a pregnant woman in French Guiana (bold) and a reference Tonate virus sequence. Tree was generated from concatenated sequences (891 bp) using a neighbor-joining algorithm. GenBank accession numbers and VEE complex subtypes are provided for reference sequences. Scale bar represents 5% nucleotide sequence divergence. VEE, Venezuelan equine encephalomyelitis.

To detect serum TONV IgM, the Arbovirus National Reference Center in French Guiana used an in-house IgM capture ELISA test that used whole virus–based antigens obtained from the brains of newborn mice and hyperimmune ascitic fluids. We calculated the ratio of the optical density obtained from the patient’s serum to the TONV antigen divided by the optical density of the same serum on a TONV-negative antigen. We set a ratio of >3 to define the presence of TONV IgM. Evolution of the test ratio from 1.1 (negative) to 19 (strongly positive) between the 2 samples with a threshold of positivity defined by a ratio >3 suggested maternal seroconversion during early pregnancy. We obtained additional molecular amplifications from amniotic fluid using primers targeting different regions of the TONV genome (Appendix Table) and sequenced the amplicons, which yielded partial genome sequences of 256 bp corresponding to the 5′NC/nonstructural protein 1 genomic region, 176 bp to the nonstructural protein 1 region, and 374 bp to the E3/E2. We compared phylogenetic analysis results of the sequences against available VEE complex sequences in GenBank, which showed that the virus was very closely related to TONV (accession no. AF075254); the considered genome sequences shared 96.8%–98.9% nt sequence identity and 98.7%–100% aa sequence identity with TONV (Figure 1; Appendix). The rarity of molecular detection of TONV and its divergence from the only strain previously available at our laboratory ruled out contamination as a possible cause of these results.

**Figure 2:**
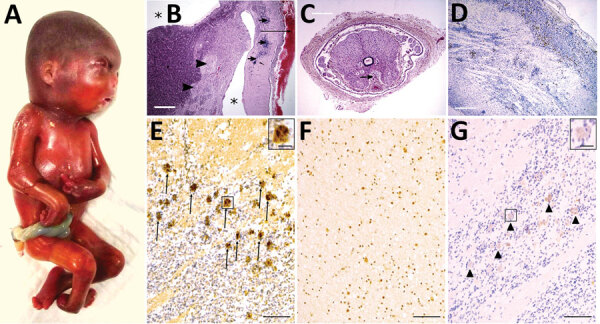
Pathologic findings including results of external examination, histological features of central nervous system, and immunohistochemical staining in a fetus from a woman in French Guiana who was found to be infected with Tonate virus. A) External examination of the body showing subcutaneous edema, microcephaly, craniofacial malformations (short forehead, flat midface), and severe arthrogryposis with upper and lower limb malformations with joint contractures. B) Histologic view of brain section stained in hematoxylin and eosin, displaying lateral ventricle enlargement (asterisk), meningeal hemorrhage (long arrow), diffuse calcifications (short arrows), and nodular heterotopia (arrowheads). Scale bar = 3 mm. C) Spinal cord section showing an abnormally shaped and atrophic spinal cord with the presence of siderophages (sign of premortem meningeal hemorrhage, arrow). Scale bar = 1 mm. D) CD68 immunohistochemistry demonstrating microglial activation and small clusters of microglia and macrophages in the brain (hematoxylin counterstain). Scale bar = 1 mm. E–G) Immunohistochemistry, using anti-TONV mouse serum, of patient (E), control (F), and negative control (G) brains. Note the strong staining of many positive cells in the patient (arrows and inset in panel E), compared to the control brain, where a moderately diffuse background signal is shown but without strong positive cells such as in the patient. In the negative control (without anti-TONV mouse serum antibody), there is a very slight staining (arrowheads and inset in panel G) in the same cells compared with those in the patient, indicating the background signal (color trapping) in these cells. Scale bars = 300 μm; insets in panels E and G = 20 μm.

Fetal autopsy identified a male fetus, small for 22 weeks of gestation, with dysmorphism and fetal akinesia deformation sequence (Figure 2, panel A). Neuropathologic examination discovered a notable meningeal hemorrhage and confirmed mild hydrocephaly (Figure 2, panel B). Histologic examination found neuronal migration disorders (overmigration and nodular heterotopia), microglial reaction, and subarachnoidal hemorrhage (Figure 2, panel D). The spinal cord was depleted of motor neurons (Figure 2, panel C). We detected multiple calcifications in the grey matter of the brain, cerebellum, upper cervical spine, and mesencephalon (Figure 2, panel B). The retina was dysplastic. In addition, the viscera revealed stigmata of ingestion of inflammatory fluid, rich in polynuclear cells. We found calcification in the liver. Because of the unavailability of a commercial probe and a positive control slide, reading the immunostaining TONV antibody test results was difficult. The high level of background suggested that the positivity of the anti-TONV signal in the cortical mantle should be interpreted with caution (Figure 2, panel E).

We report a detailed description of fetal anomalies, mainly neurological, associated with vertical transmission of TONV in the first half of an asymptomatic pregnancy. Despite a wide prevalence in the Guianese population (52.9% in the Bas Maroni region in 2001), human infections with TONV remain poorly documented, unsurprising given the scarcity of diagnostic tools in French Guiana. TONV often involves signs and symptoms described as dengue fever–like and in rare cases, encephalitis, which attest to the neurotropism of the virus (*9*,*10*). The present diagnosis became possible only through the recent implementation of real-time RT-PCR for VEE detection at the Arbovirus National Reference Center.

The evidence of vertical transmission of TONV we present could be an exception or could be more common, its occurrence having gone undetected mainly because of a lack of testing facilities. Documenting the possibility of vertical transmission of TONV by partially sequencing the viral genome in the amniotic fluid is a substantial finding indicating that the virus should be considered for public health monitoring (*11*,*12*) even though no previous cases of fetal abnormalities related to this virus have been reported. The presence of TONV in the amniotic fluid of a pregnant woman with a fetus with severe anomalies raises questions about a possible causal link that require special attention.

First, the co-occurrence of several histological features (presence of polynuclear cells in the digestive tract, intense glial reactions observed in the nervous system, and cellular calcifications) indicates a potential fetal infection with immunological reaction and cellular deaths. Viral encephalitis is a major cause of microglial activation and microglial nodules. Second, the spectrum of fetal lesions, particularly those observed in the central and peripheral nervous systems, has been observed with other neuroteratogenic viruses (*11*–*14*). Thus, microcephaly, which received broad public attention during the Zika epidemic, appears to be the common outcome of first-trimester infections with a wide range of neuroteratogens (*12*). In our observation, although the hypothesis of a genetic cause cannot be eliminated, the fact that the patient had a normal karyotype, plus results from an array of comparative genomic hybridization and 3-generation pedigree, suggest low risk that the condition resulted from a genetic disorder. Moreover, a study providing a historical description of 8 fetuses during a 1962 VEE virus outbreak observed a hemorrhagic component in VEE virus–related fetal brain damage (*2*), in line with observations of the fetus in our study, indicating stigmata of hemorrhages, both old and recent, supporting the hypothesis of an infectious origin. On the basis of findings from a series of autopsies, the VEE virus study describes a case of a first-trimester maternal infection in which the fetus manifested the same spectrum of lesions, including microcephaly, arthrogryposis, and ocular anomalies (*2*). However, although immunostaining did not yield any strong evidence for the presence of TONV in the brain, we believe that these anomalies associated with confirmed maternal seroconversion should be reported. As experienced during the 2015–2016 Zika epidemic, any delay in identifying teratogens can have serious consequences (*13*).

In conclusion, our findings illustrate the possibility of vertical transmissibility of TONV and strongly suggest its neuroteratogenic effects, even in asymptomatic women. The virus’s potential ability to spread beyond current endemic areas makes it critical that diagnostic tools become widely available to strengthen epidemiological surveillance and to provide more data about the potential danger of TONV for pregnant women.

Appendix Additional information on vertical transmission of Tonate virus in a pregnant woman in French Guiana.
