# Supplementary figures and images for: Tonate Virus and Fetal Abnormalities, French Guiana, 2019
Source: Emerg Infect Dis. 2022 Feb;28(2):445–8. doi: 10.3201/eid2802.210884 (PMC8798706; doi:10.3201/eid2802.210884)

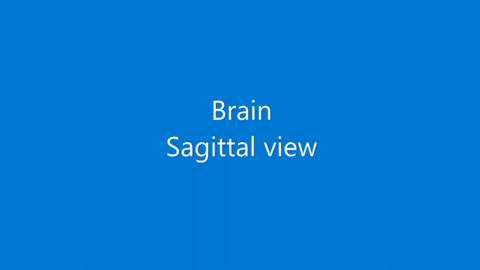

Supplement: Supplementary file 1 [file 21-0884-V.gif]
