# Supplementary material for: Tonate Virus and Fetal Abnormalities, French Guiana, 2019
Source: Emerg Infect Dis. 2022 Feb;28(2):445–8. doi: 10.3201/eid2802.210884 (PMC8798706; doi:10.3201/eid2802.210884)
Supplement: Appendix — Additional information on vertical transmission of Tonate virus in a pregnant woman in French Guiana. [file 21-0884-Techapp-s1.pdf]

Article DOI: https://doi.org/10.3201/eid2802.210884

Tonate Virus and Fetal Abnormalities, French Guiana, 2019

Appendix

Prenatal Ultrasound

Ultrasonography was performed at 20 weeks of pregnancy using a Voluson E10 scanning machine (General Electric Medical System, https://www.gehealthcare.com) equipped with a convex 2–9 MHz and a 3-dimensional, convex, 2–6 MHz volumetric transducer. From the resulting images, we compared 3 orthogonal sections of the affected fetus brain alongside corresponding sections of a normal brain (Appendix Figure) to search for anomalies (Appendix Video, https://wwwnc.cdc.gov/EID/article/28/2/21-0884-V1.htm).

Microbiological and Genetic Investigations

Karyotype and array comparative genomic hybridization (SNP Affymetrix, https://www.thermofisher.com; resolution 200 KB) on amniotic fluid were performed at Lab Cerba (https://www.lab-cerba.com). TORCH PCR or real-time PRC assays for *Toxoplasma gondii*, parvovirus B19, herpes virus simplex 1 and 2, cytomegalovirus, enterovirus, and rubeola viruses were performed at the same laboratory. A second sample of amniotic fluid was tested by mass spectrometry for oligosaccharides, glycosaminoglycans, acid hydrolases, glycosphingolipids, and sialic acid at the Centre de Biologie et Pathologie Est, Lyon Centre Université Center HCL (https://www.chu-lyon.fr/laboratoire-biologie). Maternal serum and a third amniotic fluid sample were tested at the Arbovirus National Reference Center (https://www.european-virus-archive.com) using an in-house reverse transcription (RT) PCR assay designed to detect various endemic Venezuelan equine encephalitis (VEE) viruses, including Tonate, Mucambo, and Cabassou. PCR tests for other common arboviruses in the Amazon region (dengue, Zika, chikungunya) were also performed at this laboratory. The detection of Tonate virus (TONV) IgM and IgG was performed with an immunocapture technique described elsewhere (*1*).

Fetopathological Examination

Immediately after macroscopic examination, the fetus was shipped in a cover impregnated with a 4% buffered formalin solution to the fetal pathology laboratory of Robert Debre University Hospital center (http://robertdebre.aphp.fr). We performed the autopsy 2–3 days later according to standard procedures, staining sections of all tissue samples with hematoxylin-eosin-saffron. We fixed brain and spinal cord samples in 4% formalin with 3g/L of $ZnSO_4$ solution added for a month, cut them into 6μm sections, and stained them with hematoxylin-eosin. We performed immunohistochemistry testing on brain and spinal sections using a Leica Bond Max immunostainer (Leica Microsystems, https://www.leica-microsystems.com) with Agilent 1/1000 dilution glial fibrillary acidic protein, Agilent 1/5000 dilution Vimentin, Agilent 1/5000 dilution CD68, Agilent 1/200 dilution CD3, Agilent 1/200 dilution CD20 (https://www.agilent.com), and Sigma-Aldrich 1/400 dilution Olig2 antibodies (https://www.sigmaaldrich.com), and 1/500 dilution ascitic fluid from TONV-hyperimmune mice. Sections were revealed with chromogenic substrate according to manufacturer instructions (Leica), counterstained with Mayer's hemalum then mounted in Pertex medium (https://www.pioneerresearch.co.uk). We acquired images using a Nikon DS-Fi2 camera and DS-L3 software (https://www.nikonmetrology.com).

Detection of Subtypes III and V of VEE Complex by Quantitative RT-PCR

We extracted viral RNA from serum and amniotic liquid with a QIAGEN RNeasy Mini Kit (https://www.qiagen.com) and used an in-house real-time reverse transcription (RT) PCR assay designed to detect different endemic VEE viruses, including Tonate, Mucambo, and Cabassou viruses; the real-time PCR amplifies a fragment of 126 bp in the nonstructural protein 1 (NSP1) coding region. We used the primers VEE-NSP1F (5-TGTCGGTGTGAGACGATAGT-3) and VEE-NSP1R (5-GCARCACAAGAATCCCTCGC-3), and labeled the probe with 6-carboxyfluorescein (FAM) and black hole quencher 1 (BHQ-1) VEE-NSP1P (5-FAM-TACAGGCCWGGACTGATAGC-BHQ1-3). We used an Invitrogen SuperScript III Platinum One-Step Quantitative RT-PCR System with ROX (ThermoFisher) according to manufacturer instructions. We performed the 45-cycle RT-PCR program by denaturing at 95°C for 15 sec followed by a 30 sec annealing and extension step at 55°C. If serum and negative controls all gave a negative result, we obtained a positive amplification from the amniotic fluid. We confirmed this result by an independent analysis of a new extraction.

Sequencing of TONV by RT-PCR and Amplicon Sequencing

We obtained partial genome sequences using 3 additional PCR assays and sequenced 3 amplicons using 256 bp to target the 5′ untranslated region (5′UTR)/NSP1, 176 bp to target the NSP1, and 374 bp to target the E3/E2 envelope glycoproteins coding sequences. We obtained the different amplicons from 3 independent RNA extractions. We performed PCRs using a QIAGEN OneStep RT-PCR Kit and used the same RT-PCR program we used previously. We sent amplicons to Genewiz (https://www.genewiz.com) Sanger sequencing services to perform sequencing reactions of the PCR products using the same primers that had been used for the PCR reaction (Appendix Table). For each amplicon, we used several sequencing reads with quality scores of 40–50 (provided by Genewiz) to generate the consensus sequence. After alignment, we manually checked sequences and chromatograms.

Phylogenetic Analyses

We aligned nucleotide sequences with the TONV reference sequence available from GenBank (accession number AF075254) using QIAGEN CLC Main Workbench 6.9 Beta 4, then adjusted manually. We generated the phylogenetic tree (Figure 1) using the neighbor-joining algorithm in the same software. We labeled virus strains by accession number and strain designation, followed by VEE complex subtype. Bootstrap values were indicated at nodes. Sequences were submitted to GenBank (accession numbers: MT313304, MT313305, MT313306).

References

1. Mutricy R, Djossou F, Matheus S, Lorenzi-Martinez E, De Laval F, Demar M, et al. at al. Discriminating Tonate virus from dengue virus infection: a matched case-control study in French Guiana, 2003–2016. Am J Trop Med Hyg. 2020;102:195–201. PubMed https://doi.org/10.4269/ajtmh.19-0156

2. Oberste MS, Weaver SC, Watts DM, Smith JF. Identification and genetic analysis of Panama-genotype Venezuelan equine encephalitis virus subtype ID in Peru. Am J Trop Med Hyg. 1998;58:41–6. PubMed https://doi.org/10.4269/ajtmh.1998.58.41

Appendix Table. Primers targeting the 5′ untranslated region (5′UTR)/NSP1, NSP1 and E3/E2 coding sequences of the Tonate virus genome.

Forward Primer	Sequence (5′→ 3′)	Reverse Primer	Sequence (5′→ 3′)
5′UTR/NSP1 TON1F	ATGGGCGGCGTATGAGAGAA	TON1SP3	CACTTCATCGGACAGATACAATGG
NSP1region VEE-NSP1F	TGTCGGTGTGAGACGATAGT	TON1164R	CTCTGAGTGCGGCCATTCACG
E3/E2region TON-CE F*	GAGAATTGCGAGCAATGGTC	TON8786R	CGATCTTGCCATACAGATTGTACC

*Previously published: VEE130F modified (2).

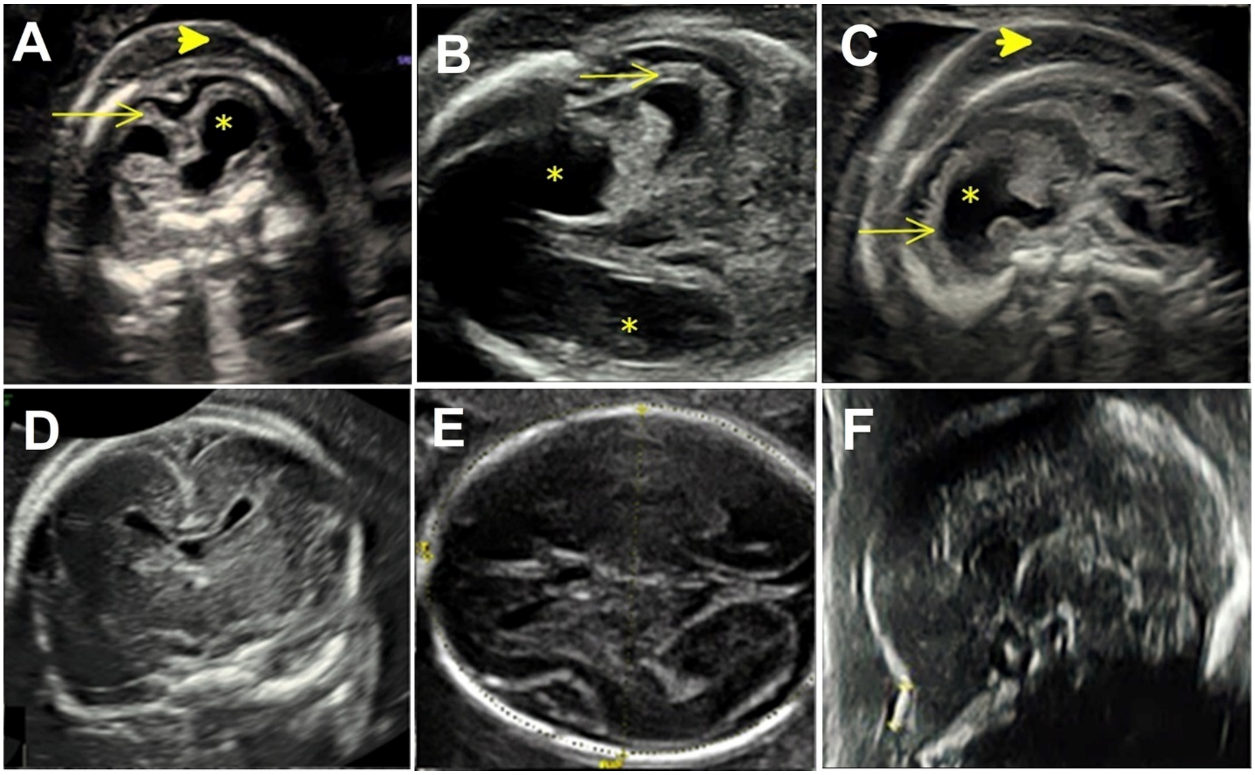

Appendix Figure. 3D fetal ultrasound images at 20 weeks of gestation using ultrasound. Affected fetal brain showed moderate ventriculomegaly (asterisk), atrophic brain mantel (arrow), subcutaneous edema (arrowhead), dysplasia of A) corpus callosum, B) brainstem, and C) cerebellum. Corresponding images of normal fetal brain development at the same stage of pregnancy in the D) corpus callosum, E) brainstem, and F) cerebellum.
